# Kinetic Modeling and Degradation Study of Liquid Polysulfide Resin-Clay Nanocomposite

**DOI:** 10.3390/molecules26030635

**Published:** 2021-01-26

**Authors:** Mohamadreza Shakiba, Arash Kakoei, Iman Jafari, Erfan Rezvani Ghomi, Mohammadreza Kalaee, Davood Zarei, Majid Abdouss, Saeid Shafiei-Navid, Fatemeh Khosravi, Seeram Ramakrishna

**Affiliations:** 1 Department of Chemistry, Amirkabir University of Technology, Tehran 15875-4413, Iran; rezashakiba011@gmail.com (M.S.); phdabdouss44@aut.ac.ir (M.A.); 2 Department of Polymer and Chemical Engineering, South Tehran Branch, Islamic Azad University, Tehran 15847-43311, Iran; a_kakouei@yahoo.com (A.K.); d_zarei@azad.ac.ir (D.Z.); 3 Department of Civil and Environmental Engineering, Faculty of Engineering, National University of Singapore, Singapore 117576, Singapore; iman.jafari@u.nus.edu; 4 Center for Nanotechnology and Sustainability, Department of Mechanical Engineering, National University of Singapore, Singapore 117581, Singapore; fatemeh_khosravi22@yahoo.com; 5 Nanothecnology Research Centre, Tehran South Branch, Islamic Azad University, Tehran 15847-43311, Iran; 6 Department of Organic Chemistry, Faculty of Chemistry, University of Mazandaran, Babolsar 47416-95447, Iran; saeed70navid@gmail.com

**Keywords:** liquid polysulfide resin, clay nanoparticle, thermal properties, nanocomposite, thermal degradation, modeling

## Abstract

Kinetic modeling and degradation study of liquid polysulfide (LPS)/clay nanocomposite is possible through Ozawa–Flynn–Wall (OFW) and Kissinger methods. Comparing the results of these models with experimental data leads to provide an accurate degradation kinetic evaluation of these materials. To this aim, the morphology and distribution of clay nanoparticles (CNPs) within the LPS matrix were investigated using Field Emission Scanning Electron Microscopy (FESEM) and X-ray diffraction (XRD). To evaluate the interaction between the LPS and the CNPs, the Fourier transform infrared (FTIR) identification was utilized. Furthermore, to investigate the kinetics of degradation, the thermal gravimetric analysis (TGA) and derivative thermogravimetry (DTG) of the samples were used in the nitrogen atmosphere with the help of Kissinger and Ozawa–Flynn–Wall (OFW) models. The characterization results confirmed the homogenous dispersion of the CNPs into the LPS matrix. In addition, the presence of CNPs increased the thermal stability and activation energy (E_a_) of the samples at different conversion rates. Moreover, the OFW method was highly consistent with the experimental data and provided an appropriate fit for the degradation kinetics.

## 1. Introduction

In 1840, polysulfide was discovered by the reaction of ethylene dichloride and potassium sulfide [1]. Eighty-five years later, polysulfide elastomers were synthesized based on 1,2-dichloroethane and sodium polysulfide in sodium hydroxide solution [2]. However, due to significant bad odors and the production of toxic and dangerous gases (e.g., hydrogen sulfide and carbon disulfide) during the synthesis of these polymers, polysulfide has been produced based on Bis(2-chloroethyl) formal since 1940 [2,3]. The most important properties of polysulfide-based polymers are their resistance to most solvents, oils, vapors, gases, light, and ozone, which allow them to be used in applications such as aircraft fuel tank sealants, printer rolls, and modifiers in the rubber industry [4,5,6,7]. Polysulfide polymers are available as crude rubber, suspended material, and liquid [8,9]. In the manufacturing of polysulfide resins, high-weight elastomeric polysulfides are first synthesized and then depolymerized to become liquid resins with a proportional molecular weight [8,10,11]. Unlike natural rubber, polysulfide polymers are not processable in rubber mills. Polysulfides show low thermal stability, and in the industry, they are commonly referred to by the brand name Thiokol, which its liquid form has the most application among polysulfides [12]. Approximately 94% of LPS are used as sealants, and about 75% of these sealants are used in doors and windows sealants in the construction industry [8,13].

However, the low thermal stability of polysulfide polymers is one of their crucial weak points [14,15,16]. Limited research has been conducted on the use of nanoparticles to improve the thermal and mechanical properties of polysulfide resins. Guchhait et al. examined the morphology of polysulfide/silica nanocomposites [7]. Their results showed that the rigid structure of polysulfide resin becomes brittle with the addition of nano-silica. In another study, the thermophysical properties of polysulfide nanocomposites containing graphene particles have been investigated by TGA and thermal differential scanning calorimetric (DSC) [9,13]. It was proven that the increase of graphene nanoparticles has a significant effect on the crystallization, glass temperature, and thermal degradation of the resulting compound. It was also found that the thermal degradation of the resulting nanocomposite is affected by the presence and the concentration of graphene particles. Compared to conventional fillers, clay nanoparticles (CNPs) present excellent characteristics due to their high aspect ratio, natural availability, and low cost [7,17,18]. More notably, throughout the composition of polymer/clay, clay can be divided into nanometer-particles, thereby preventing the agglomeration issue that usually occurs in the production of other nanocomposites made up of polymer and nanofillers [19]. In this regard, polysulfide/CNPs nanocomposites have been investigated in some studies. Macadam et al. observed that when CNPs were introduced to sunflower oil-based polysulfide, the glass temperature and the thermal resistance increased [10]. Pradhan et al. have studied the effect of CNPs on the adhesion and mechanical properties of LPS [17]. They concluded that the strength of the cured polysulfide structure increases significantly with an increase of CNPs amount.

However, based on the early literature studying the thermal behavior of polysulfide/CNPs nanocomposites, there is a lack in the study of the degradation kinetics with the view of comparing and matching different modeling methods with experimental results. Methods including Ozawa–Flynn–Wall (OFW), Friedman, Kissinger, and Coats-Redfern are widely utilized in determining the parameters of thermal degradation [18]. Some of these methods, such as OFW and Kissinger, can be applied to study the kinetics degradation of polymer nanocomposites using the results of TGA and DSC analyses [20,21]. Therefore, in the current study, in addition to the fabrication and characterization of LPS/CNP nanocomposites, the modeling of the thermal degradation behavior was performed by Kissinger and OFW methods. Furthermore, proportional fitting of degradation kinetics due to the presence of the CNPs in different amounts up to 5 wt.% are provided.

## 2. Materials and Methods

### 2.1. Materials

LPS was purchased from Troy Company (Hanover, Germany) with the specifications mentioned in Table 1. Manganese dioxide and (MnO_2_) (CAS # 1313-13-9; molecular weight: 86.94; particle size: 200 mesh; crystalline powder; manganometric ≥ 89.0%) and diphenyl guanidine (DPG) (CAS # 102-06-7; molecular weight: 211.26; white to pale pink powder 97%) were obtained from Kimia Sazan company (Tehran, Iran). The type of CNPs used was Closite 30B, produced by Southern Clay Company (Louisville, KY, United States). The Closite 30B is a montmorillonite modified quaternary ammonium salts.

### 2.2. Fabrication of LPS/CNPs Nanocomposite

First, CNPs were placed in an oven at 80 °C for 4 h to remove absorbed moisture. Then, 20 g of LPS along with CNPs according to Table 2 were poured into a beaker and stirred with a homogenizer for 15 min at a speed of 5000 rpm. Subsequently, 0.2 g of DPG as an accelerator was added to the system, and the homogenization continued at the same rate for 10 more minutes to disperse the CNPs well into the LPS matrix. In the next step, 3 g of MnO_2_ as a curing agent was added to the mixture, and mixing was continued through the homogenizer for another 5 min. During the mixing process, the mixing container was placed in an ice bath to hinder the increase in temperature and prevent the pre-curing of the resin. Then, the prepared resins were put in a sealed container, and the air inside it was removed using a vacuum pump. Next, the resins were quickly poured into a silicone mold with a thickness of 500 microns using a film applicator. Finally, to complete the samples’ curing process, the silicone molds were aged at ambient temperature for one day and then were placed in an oven at 90 °C for 4 h [22].

### 2.3. Characterization

To investigate the morphology of the fabricated LPS/CNPs nanocomposite samples, FESEM studies were performed using Oxford Instruments (INCA) equipped with Energy Dispersive X-ray (EDX) analyzer. In addition, the XRD test for all samples, including the neat CNPs, was performed using Analytical Diffractometer (PW1800) using CuKα radiation with a voltage of 40 kV and a current of 30 mA. Moreover, FTIR spectroscopy was conducted via a Vertex 80 device (Bruker, Karlsruhe, Germany) to ensure the successful preparation of LPS/CNPs nanocomposites. To investigate the thermal degradation of the samples, TGA measurements (Perkin-Elmer STA 6000, Waltham, MA, USA) were used at heating rates of 10 °C/min, 15 °C/min, and 20 °C/min under a nitrogen atmosphere.

### 2.4. Kinetic Analysis Techniques

Different techniques can be used to explore the thermal behavior of materials, such as TGA, DTG, DSC, and differential thermal analysis (DTA) [23,24]. Evaluating the data obtained in these techniques provides significant information about the reactivity and stability of materials based on the kinetic analysis. The Kissinger method is one of the most popular kinetic analysis techniques that can be used in thermally stimulated processes to estimate and evaluate the E_a_ through an unparalleled simple way [25].

According to an article by Kissinger [26], Equation (1) was obtained based on the Arrhenius theory in which the rate constant depends on the temperature, and the reaction order model corresponds to the conversion function.

(1) $$\mathrm{Ln}\frac{\beta }{T_{m}^{2}}\;=\;\ln\frac{AR}{E_{a}}\;+\;\ln[n{\left(1-\alpha_{m}\right)}^{n-1}]\;-\;\frac{E_{a}}{RT_{m}}$$

where β is the heating rate, A is the pre-exponential factor, E_a_ is the activation energy, n is the reaction order, α is the conversion degree, T_m_ is the maximum weight loss temperature, and R is the universal gas constant. For a given value of n, the component ln[n(1 − α_m_)^n−1^] is constant, and consequently, E_a_ can be obtained by estimating the slope of the plot $\ln\left(\frac{\beta }{T_{m}^{2}}\right)$ vs. $\left(\frac{1}{T_{m}}\right)$.

The OFW method is also one of the methods that can be used to estimate the E_a_ of the heat degradation reaction [27]. In this method, it is presumed that for all conversion values (α), the conversion function f(α) would not change by changing the heating rate. Temperatures corresponding to the percentage of constant conversion (α) are often calculated at various heating rates (β), and the plot of ln (β) versus $\left(\frac{1}{T}\right)$ is finally produced. The relationship is described as an equation in this technique (Equation (2)):

(2) $$\ln\left(\beta \right)=\ln\left(\frac{\mathrm{Af}\left(\alpha \right)}{d\alpha /\mathrm{dT}}\right)-\frac{\mathrm{Ea}}{\mathrm{RT}}$$

where A is the projection coefficient that is considered independent of temperature, E is the activation force, T is the absolute temperature, and R is the fundamental gas constant. The plot of ln(β) versus $\left(\frac{1}{T}\right)$ gives a straight line, from the slope of which it is possible to obtain the E_a_.

Kissinger and OFW methods are model-free analyses that estimate the E_a_ without considering a kinetic model for a reaction process. In these analyses, it is not required to determine the reaction type in which the activation energy should be estimated. These methods are integral and iso-conversional and require positive heating rates. In addition, these methods can be used in multiple-step reactions without parallel reaction steps and can evaluate each reaction point individually. Using these methods, the thermal kinetic parameters that characterize the thermal degradation process can be estimated. Hence, these widely known mathematical models were used in this study to calculate the activation energy of the thermal degradation process for LPS and LPS/CNPs [28].

## 3. Results and Discussion

**Figure 1:**
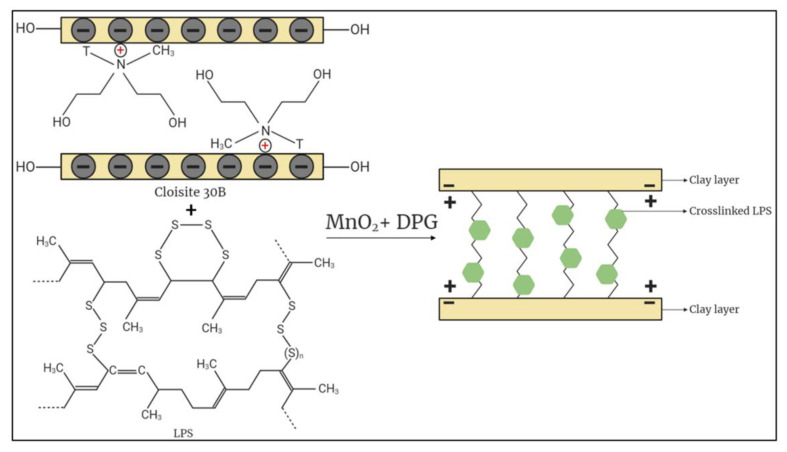
An illustration of the structure of liquid polysulfide/clay nanoparticles (LPS/CNPs) nanocomposite.

It is well-known that the incorporation of highly dispersed CNPs helps with enhancing polymeric matrices properties, and an illustration of the cured LPS/CNPs structure intercalated within the gallery regions of the CNPs is shown in Figure 1.

### 3.1. Morphological Assessment

**Figure 2:**
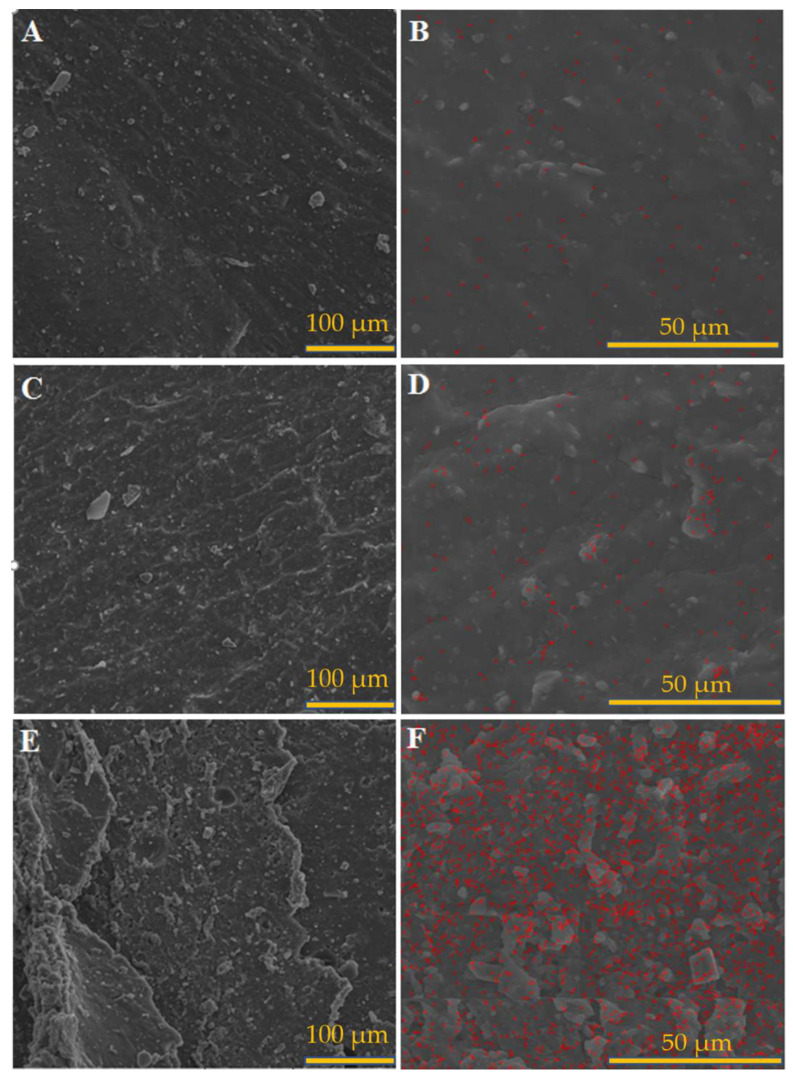
FESEM images for (**A**) LPS/CNPs 1%, (**C**) LPS LPS/CNPs 3%, (**E**) LPS/CNPs 5%, and the distribution map for Si element for (**B**) LPS/CNPs 1%, (**D**) LPS LPS/CNPs 3%, (**F**) LPS/CNPs 5%.

The FESEM-EDX micrographs of the LPS/CNPs 1%, LPS/CNPs 3%, and LPS/CNPs 5% samples are shown in Figure 2. The distribution maps of the Si element (Figure 2A,C,E) indicate that CNPs have a uniform distribution in the LPS matrix. Good compatibility and interaction between the CNPs and LPS matrix led to a high level of distribution with no sign of agglomeration for all samples, and with a higher amount of CNPs, the marked points were also augmented.

### 3.2. Structural Characterization

**Figure 3:**
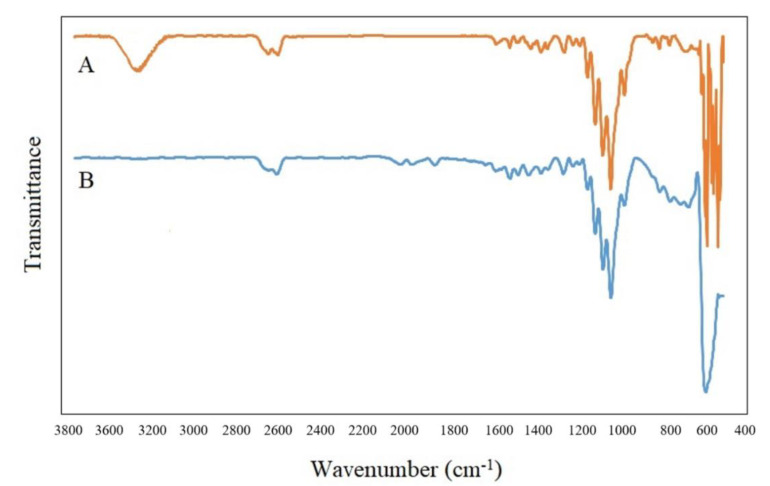
FTIR spectra of (A) neat LPS and (B) LPS/CNPs 3%.

The FTIR spectra of neat LPS and LPS/CNPs 3% are presented in Figure 3. By comparing the two spectra of neat LPS and LPS/CNPs 3%, it was found that there are three absorption bands in the LPS matrix at 2185, 2120, and 1990 cm^−1^ that have been removed in nanocomposites, and this could be due to the good interaction between sulfide groups and CNPs. The broad absorption band at 3600 cm^−1^ corresponds to the -OH groups of the CNPs, and the bands in the region of 500 cm^−1^ to 600 cm^−1^ suggest the C-Cl bond and are attributed to the curing agent.

**Figure 4:**
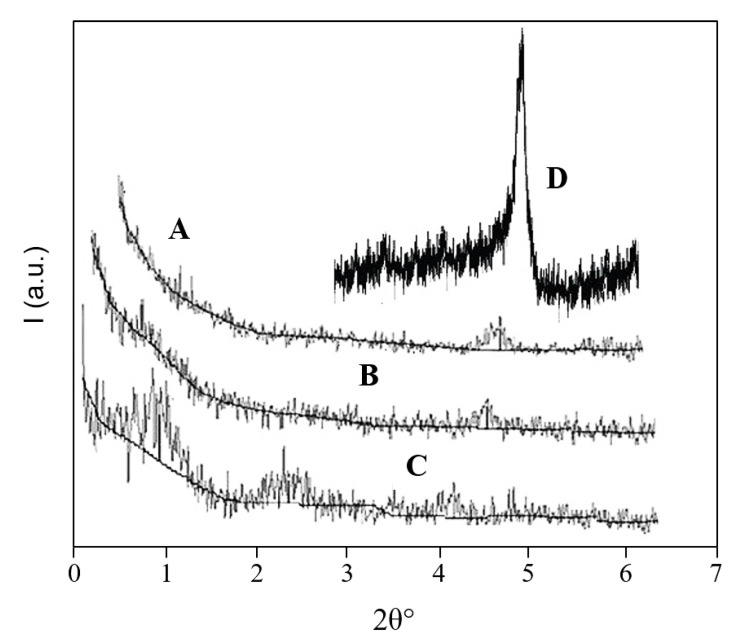
Results of XRD for samples: (**A**) LPS/CNPs 1%, (**B**) LPS/CNPs 3%, (**C**) LPS/CNPs 5%, and (**D**) neat CNPs.

Further, XRD was used to characterize the crystallinity level of the samples. Figure 4 displays the XRD patterns of the LPS/CNPs 1%, LPS/CNPs 3%, and LPS/CNPs 5% samples in the range of 2θ = 0–7°. One reflection at 2θ = 5.1° was represented by the XRD pattern of the CNPs (Figure 4), which measured the interlayer distance (*d*-value) about 20 A° according to Bragg’s equation and showed the crystallinity of the structure. The peak intensity at 2θ = 5.1° was sharp for the neat CNPs, which decreased due to the introduction of CNPs in the LPS matrix, and the crystal structure was no longer visible easily. The change in the crystallinity of the samples occurred possibly for two reasons: first, due to the changes in the layered structure of CNPs after homogenizing with the LPS matrix, and second, because of the good distribution of nanoparticles in the LPS matrix and the lack of agglomeration [29].

### 3.3. Thermal Properties

**Figure 5:**
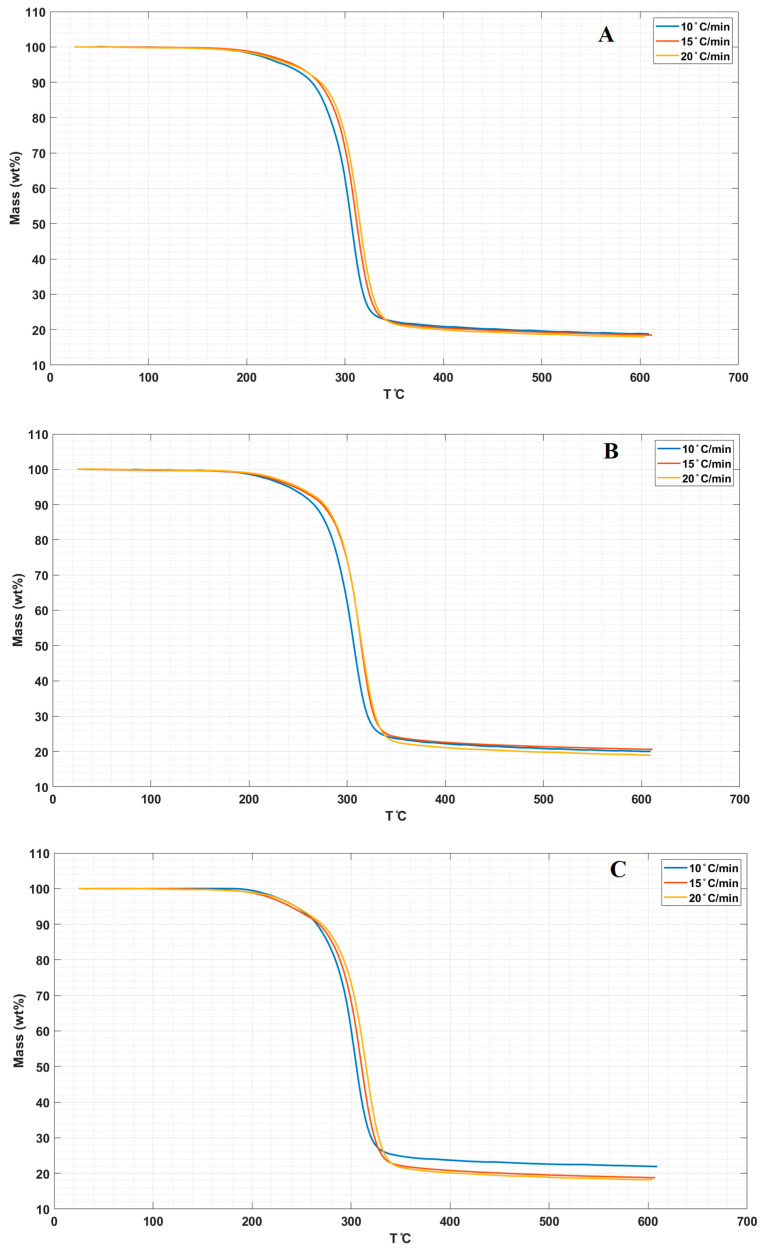

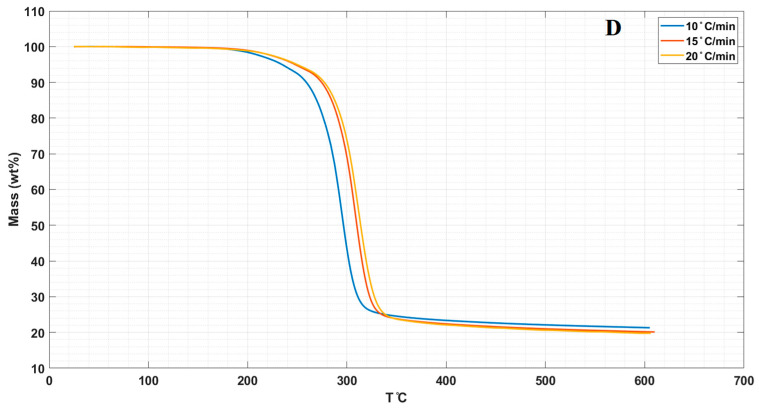
TGA curves in 3 different heating rates for (**A**) LPS, (**B**) LPS/CNPs 1%, (**C**) LPS/CNPs 3%, and (**D**) LPS/CNPs 5%.

(Figure 5A–D) display the TGA curves for the neat LPS and LPS/CNPs nanocomposites in three different heating rates of 10 °C/min, 15 °C/min, and 20 °C/min.

In addition, Table 3 shows the relevant data obtained from TGA analyses for neat LPS and the LPS/CNPs nanocomposites.

Comparing the results for the neat LPS and LPS/CNPs nanocomposites in three heating rates revealed the fact that LPS/CNPs nanocomposites showed higher thermal resistance than the neat LPS matrix. All thermal temperatures were increased by increasing the heating rates from 10 °C/min to 15 °C/min and then 20 °C/min. T_0.1_, T_0.5_, and T_m_ temperatures in the LPS/CNPs 5% were decreased in all heating rates compared to the LPS/CNPs 3%, except in T_0.5_ and T_m_ related to the heating rate of 20 °C/min. These reductions in mass loss temperatures for LPS/CNPs 5% can be compared to the destructive effect of active free radicals due to catalytic properties or possible agglomeration by a higher amount of CNPs in the samples [7]. The increase in the temperature of the heat degradation of nanocomposites containing lower percentages of CNPs than 5% can be justified by the decrease in the free movement of volatile products in polymer nanocomposites due to the heat degradation (gases emitted during sample pyrolysis) [17,30]

In general, it can be observed from Table 3 and Figure 5 that the inhibitory effect of CNPs increases the thermal stability of the final nanocomposites, which is typically the primary effect at low clay concentrations. The thermal stability of the samples at low percentage clay incorporation is due to the proper interaction between the CNPs and the LPS matrix as shown in Figure 1, as well as the shielding effect of the charred polymer on the surface, which stops oxygen from being widely absorbed into the polymer matrix and raises the temperature of degradation [21,31].

### 3.4. Determination of Degradation Kinetic Parameters

**Figure 6:**
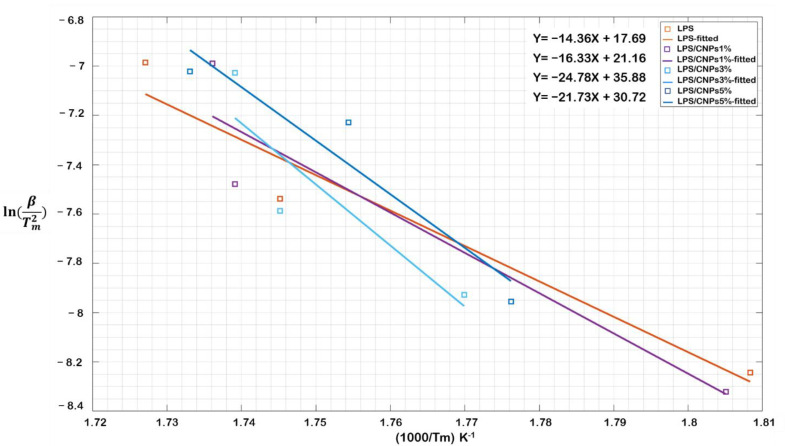
Kissinger’s relationship chart for LPS and nanocomposites.

In this study, the methods of Kissinger and OFW were used to estimate E_a_ for thermal degradation of neat LPS and LPS/CNPs nanocomposites. To estimate the E_a_ of heat degradation, the Kissinger method equation was obtained at the T_m,_ in which the first derivative weight loss is equal to zero (dw/dt = 0). In TGA, measurements at a constant heating rate, time, and temperature derivatives of weight loss rates are linearly correlated, and the data can be plotted as a function of time or temperature and then can be analyzed using Kissinger’s method. Based on the data obtained from the TGA profile, the plots of $\ln\left(\frac{\beta }{T_{m}^{2}}\right)$ vs. $\left(\frac{1}{T_{m}}\right)$ for neat LPS and LPS/CNPs nanocomposites are shown in Figure 6. The E_a_ can then be obtained from the slope of the corresponding fitted lines.

As shown in Figure 6, the slope of all fitted lines is negative, including neat LPS and LPS/CNPs nanocomposites. Table 4 shows the E_a_ and correlation coefficient (R^2^) for all samples obtained from Kissinger’s method. Accordingly, by adding 1% of CNPs to LPS, E_a_ increased from 119 kJ for neat LPS to 136 kJ. By a further increase in CNPs concentration, the E_a_ reached 206 KJ/mol for LPS/CNPs 3%. However, by adding the CNPs from 3 to 5wt.%, the E_a_ dropped significantly due to the destructive effect of active free radicals. The pattern of these observations indicates that the E_a_ improved by increasing the amount of CNPs in the samples up to 3 wt.% and before the catalytic effect domination. In other words, without considering the effect of active free radicals in a specific range of CNPs concentration and with the inclusion of CNPs, the rate of thermal degradation of samples decreased, and consequently, the thermal stability of samples containing CNPs improved compared to neat LPS.

**Figure 7:**
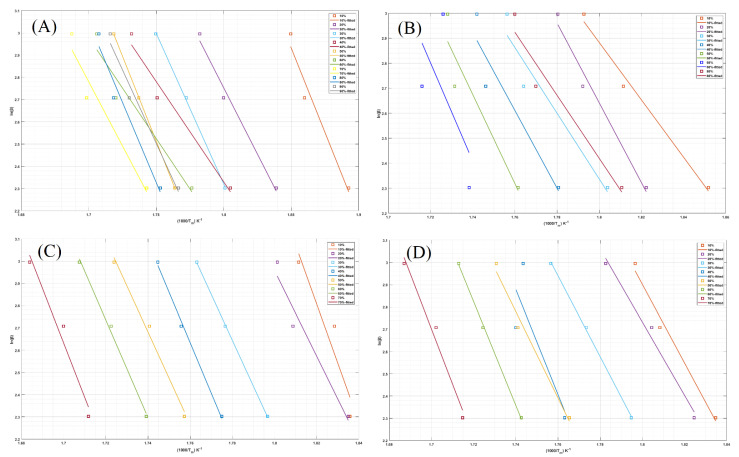
Ozawa–Flynn–Wall (OFW) drawing for (**A**) LPS, (**B**) LPS/CNPs 1%, (**C**) LPS/CNPs 3%, and (**D**) LPS/CNPs 5%.

Another technique based on the OFW method was also used in this study to model the thermal degradation kinetics and estimate the E_a_ of the samples. The plots of ln(β) versus $\left(\frac{1}{T_{m}}\right)$ for neat LPS and LPS/CNPs nanocomposites based on the data obtained from the TGA are shown in Figure 7, and all fitted lines have a negative slope. At constant conversion rates of 10 to 90%, LPS/CNPs nanocomposites demonstrated a reasonably good linear relationship, suggesting that the OFW analysis is a good method in the explanation of these nanocomposites’ thermal degradation kinetics based on TGA analysis results. The results of this study were plotted for LPS/CNPs nanocomposites in addition to the neat LPS at a constant conversion rate (from 10 to 90%) in the form of ln(β) vs. $\left(\frac{1}{T}\right)$. Except for a few cases, these diagrams illustrate that the lines derived from this process are parallel to each other and that the E_a_ can be determined for each CNPs percentage by measuring the average slope of all corresponding fitted lines.

**Figure 8:**
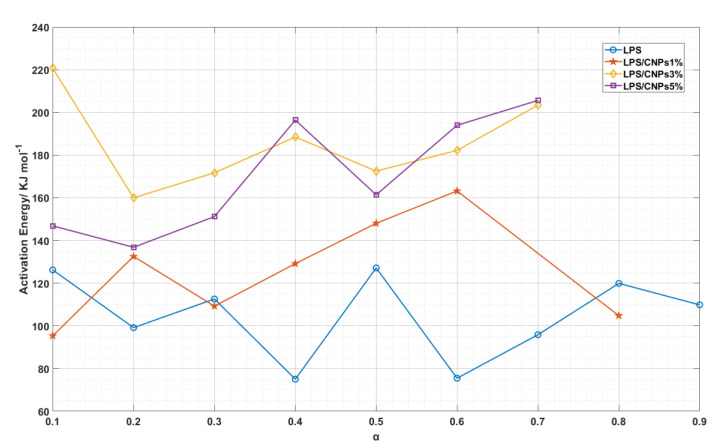
Variation of activation energy of neat LPS and LPS/CNPs nanocomposites versus conversion percentage.

Figure 8 displays a diagram of the activation energies obtained in terms of the conversion percentage, and the E_a_ varies with an increase in the rate of conversion. In compliance with Figure 8, for neat LPS and LPS/CNPs nanocomposites, the average sum of E_a_ is highly consistent with the Kissinger process’s corresponding value.

Table 5 presents the E_a_ of the thermal degradation reaction of neat LPS and the LPS/CPNs nanocomposites measured by the OFW method. According to Table 5, the average E_a_ of LPS/CPNs nanocomposites were higher than neat LPS. This also reveals that the E_a_ increased with an increase in the concentration of CNPs to 3 wt.%, and then decreased by increasing the percentage of CNPs to 5 wt.%.

The reduction in E_a_ for LPS/CNPs 5% is due to the catalytic effect, which stems from the destructive activity of active macroradicals. The good interaction between CNPs with the LPS matrix as well as the blockage of volatile gas produced by the thermal decomposition of the LPS structure is the source of improving thermal stability in LPS/CNPs nanocomposites. Thermal decomposition begins from the surface of the nanocomposites according to the barrier model [32], which results in an increase in the amount of CNPs in the degrading nanocomposite and the forming of a protective layer on the surface of the matrix where oxygen prevents the matrix from expanding under this layer. Newly shaped radicals arising from the degradation of the polymer are also trapped by the CNPs surfaces according to the nano-amplification theory [33], and a sequence of intermolecular reactions will occur. The CNPs steadily move to the surface as the degradation process continues, forming a surface barrier due to the reduction of surface energy [10]. In addition, from the findings of Table 5 (larger R^2^ values and OFW analyses), the superiority of the OFW method in explaining the thermal degradation of nanocomposites relative to the Kissinger method is clear. In other words, improving the thermal stability of LPS-CPNs nanocomposites depends on the development and stabilization of CPN bonded macro radicals. Different effects of the presence of CNPs can result in a considerable increase in thermal stability, which results in a decrease in diffusion, producing ash as a matrix defender on the CNPs surface, slowing down the escape of volatile materials by CNPs during decomposition, and absorption of gases released by the pyrolysis effect by CNPs.

In either case, the low percentage incorporation of CNPs (up to 3 wt.%) with an inhibitory effect postpones the degradation of samples and increases their E_a_. On the other hand, the catalytic effect was prominent in LPS/CNPs nanocomposites with 5 wt.% incorporation of CNPs, the increase in E_a_ proportionately decreased, and the thermal activity was also impaired by agglomeration and poor distribution of nanoparticles [34].

## 4. Summary

In this study, LPS/CNPs nanocomposites were prepared, and the presence of CNPs and their interactions with the LPS matrix were assessed via FTIR, XRD, FESEM, and EDX analyses. The results of the FESEM-EDX images confirmed the good distribution of CNPs in the LPS matrix. The TGA and DTG analyses were used to model the thermal degradation of the neat LPS as well as the LPS/CNPs nanocomposites using Kissinger and OFW methods. The results of this study showed that the thermal stability and the increase in E_a_ of the samples were caused by the presence of CNPs with high compatibility with the polymer matrix. On the other hand, while the slope of fitted lines in both Kissinger and OFW modeling methods was approximately the same, the OFW approach offered a better fit for the sample’s degradation kinetics.

## Figures and Tables

**Table 1:** Technical properties of used polysulfide.

Appearance	Content SH (%)	Viscosity (pa.s)	Average Molecular Weight (g/mol)	Sulfur Content (%)
Brown liquid	5–7	1.3	1100	37–38

**Table 2:** The composition of LPS and LPS/CNPs nanocomposites.

Samples	LPS (g)	DPG (g)	MnO_2_ (g)	CNP (g)
LPS	20	0.2	3	0
LPS/CNPs 1%	20	0.2	3	0.2
LPS/CNPs 3%	20	0.2	3	0.6
LPS/CNPs 5%	20	0.2	3	1

**Table 3:** Thermal properties data of LPS and LPS/CNPs nanocomposites.

10 °C/min			
Samples	T_0.1_(°C) ^a^	T_0.5_(°C) ^b^	T_m_ (°C) ^c^
LPS	252	290	280
LPS/CNPs 1%	256.7	294.5	281
LPS/CNPs 3%	258.7	297.9	292
LPS/CNPs 5%	257	295	290
**15 °C/min**			
**Samples**	**T_0.1_(**°C**) ^a^**	**T_0.5_(°C) ^b^**	**T_m_ (°C) ^c^**
LPS	258.5	300.5	300
LPS/CNPs 1%	264.9	302.4	302
LPS/CNPs 3%	267.9	301.3	300
LPS/CNPs 5%	265	300.5	297
**20 °C/min**			
**Samples**	**T_0.1_(°C) ^a^**	**T_0.5_(°C) ^b^**	**T_m_ (°C) ^c^**
LPS	264.5	304.7	306
LPS/CNPs 1%	268.8	306.5	303
LPS/CNPs 3%	269.5	306.9	302
LPS/CNPs 5%	268.6	308.6	304

^a^ Temperature at 10% mass loss; ^b^ temperature at 50% mass loss; ^c^ maximum mass loss temperature was obtained from DTG thermograms.

**Table 4:** The E_a_ and the correlation coefficient calculated by the Kissinger method.

R^2^	E_a_ (Kj/mol)	Samples
0.9712	119	LPS
0.9451	136	LPS/CNPs 1%
0.8888	206	LPS/CNPs 3%
0.9542	181	LPS/CNPs 5%

**Table 5:** The E_a_ and the correlation coefficient calculated by the OFW method.

R^2^	E_a_ (Kj/mol)	Samples
0.9859	105	LPS
0.9198	126	LPS/CNPs 1%
0.9859	186	LPS/CNPs 3%
0.9728	170	LPS/CNPs 5%

## Data Availability

The data presented in this study are available on request from the corresponding author.

## Sample Availability

Samples of the compounds are not available from the authors.
